# A Multirate Control Strategy to the Slow Sensors Problem: An Interactive Simulation Tool for Controller Assisted Design

**DOI:** 10.3390/s140304086

**Published:** 2014-02-27

**Authors:** Julián Salt, Ángel Cuenca, Francisco Palau, Sebastián Dormido

**Affiliations:** 1 Department of Systems and Control Engineering, Universitat Politecnica de Valencia, Camino de Vera s/n, 46022 Valencia, Spain; E-Mails: acuenca@isa.upv.es (A.C.); fraparo@inf.upv.es (F.P.); 2 Department of Computer Science and Automatic Control, UNED, 28040 Madrid, Spain; E-Mail: sdormido@dia.uned.es

**Keywords:** slow sensors, non-uniform sampling, multirate control, interactive simulation, control education, non-uniform multirate controllers

## Abstract

In many control applications, the sensor technology used for the measurement of the variable to be controlled is not able to maintain a restricted sampling period. In this context, the assumption of regular and uniform sampling pattern is questionable. Moreover, if the control action updating can be faster than the output measurement frequency in order to fulfill the proposed closed loop behavior, the solution is usually a multirate controller. There are some known aspects to be careful of when a multirate system (MR) is going to be designed. The proper multiplicity between input-output sampling periods, the proper controller structure, the existence of ripples and others issues need to be considered. A useful way to save time and achieve good results is to have an assisted computer design tool. An interactive simulation tool to deal with MR seems to be the right solution. In this paper this kind of simulation application is presented. It allows an easy understanding of the performance degrading or improvement when changing the multirate sampling pattern parameters. The tool was developed using Sysquake, a Matlab-like language with fast execution and powerful graphic facilities. It can be delivered as an executable. In the paper a detailed explanation of MR treatment is also included and the design of four different MR controllers with flexible structure to be adapted to different schemes will also be presented. The Smith's predictor in these MR schemes is also explained, justified and used when time delays appear. Finally some interesting observations achieved using this interactive tool are included.

## 1. Introduction

A multirate sampling (MR) system is defined as a hybrid system composed of continuous time elements, usually the plant, and some discrete time components, usually the controllers or the filters, where two or more variables are sampled or updated at different frequencies. It can be also considered that the discrete actions are not equally spaced on time and/or delayed. Moreover, in a great number of computer control applications the approximation of a regular pattern of sampled signals is assumed.

A non-very restrictive assumption to simplify the treatment is to consider that the sampling pattern is periodic. That is, the process variables are sampled and/or updated at different and/or irregular intervals, but there is a global period *T*_0_ with cyclic repetition. It may be also considered that there is a delay between the sampling and the updating of variables, but a global periodicity is still assumed. The case of asynchronous sampling/updating, with a random occurrence of the discrete actions, is much more complicated and it will not be considered in this paper.

In a basic digital control system, a perfect uniform sampling and updating pattern of the involved variables is assumed, but it should be pointed out that, in practical applications, the synchronicity of the set of discrete actions is not perfect or it can be modified in order to improve the performances. Thus, MR is an important issue not only for research purposes but also from a practical point of view. MR may be present in a wide range of applications and the users must understand its consequences in an easy way. Chemical analyses, or samples obtained by artificial vision with post-processing requirements need a time interval that for a real-time process control request could be long. Some other similar problems appear when the sensors are spatially separated from the controller algorithm device: distillation columns, UAVs, network based control schemes, *etc*.

The control target is to achieve similar performances to those the faster single rate controller would provide. However, in these cases, the theoretical analysis of the controlled system performances is much more computationally involved. The modeling, analysis and design steps can consume a great amount of engineering time.

In order to analyze and study the different characteristics of the dynamic behavior of a MR, it is common to use time and frequency techniques and tools. These will provide a complete and global picture of the system behavior, showing up the interrelation among the different controlled plant performances.

The combined use of control system design tools and dynamic system simulation, leads to the computer aided control system design environments, simplifying the design task. Additionally the possibility of simultaneous visualization in various windows of the effects in different performances of some design parameter changes helps to observe with more flexibility the change gradient over the system [1]. This facility provides the understanding of the usual steps in a design procedure. The perception of synthesis and analysis phases is simultaneous with the consequent effort saving with relation to classical simulation environments. In general, the complexity of the theoretical developments justifies the use of interactive simulation techniques that also allow for acting over a high number of parameters with hard crossed relations. The global and simultaneous dynamic visualization of different kinds of time and frequency diagrams allows grasping a clear understanding about the effects of the concerned topic [2].

In this sense some years ago, Åström and colleagues at the Lund Institute introduced some valuable concepts for control education task aid. In this context the significance of concepts like dynamic pictures and virtual interactivity must be highlighted. This original idea was implemented in packages as Ictools and CCSdemo, that Johansson *et al.* [3] and Wittenmark *et al.* [4], developed at the Department of Automatic Control at the Lund Institute of Technology, and Sysquake, developed at the Institut d'Automátique of the Federal Polytechnic School of Laussanne by Piguet [5,6]. The old use of computer aided control systems design was definitively improved. The dynamic picture allowed one to handle with the mouse a set of different nature graphic windows with some common parameter/s among them. Some change in a parameter manipulated by the user implied the fast—practically immediate—modified visualization in graphics influenced by that object. One of the main advantages is that the user does not need the implementation of code sentences. The complete effort is leading to testing and understanding of the system control ideas and principles that the application involves.

In the MR case this kind of application appears indispensable. Some key concepts in order to model, analyze and design MR systems are overcome by the use of this interactive application. The working principles of MR are easily understood using this procedure. A specific Sysquake application was implemented for MR. This tool takes advantages of the fast execution and excellent graphic features that the use of Sysquake provides. In summary, the main motivations in writing of this paper are the following: (1) to study, in a very direct way, how the modification of the different sampling periods that are involved in a MR system can lead to unexpected behaviours; (2) to present in a unified way the performance of different MR controllers in order to choose by means of this tool the most appropriate design in each case; (3) to provide a tool that is not only useful to do research on this kind of systems, but also to improve the learning of MR concepts for beginners.

The structure of the paper is as follows: the next section introduces some preliminary material and foundations, definitions and notation; Section 3 presents the problem statement and provides the detailed design procedure of the assumed controllers. Section 4 describes the developed application using interactive simulation techniques, and then some interesting examples derived with this tool are given in Section 5. A conclusion section closes the paper.

## 2. Theoretical Foundations

**Figure 1.**
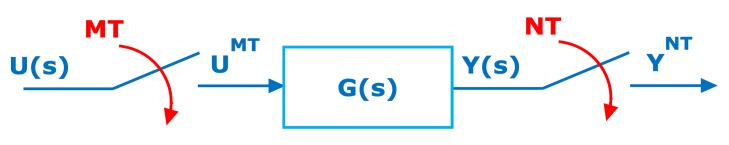
An initial MR System.

The scope and purpose of this work has been exposed in the previous section. In this section, the general problem, basic notations and operations among signals and processes are going to be introduced. After exposing the kind of problems that a practitioner finds when consider this topic, the elemental signal change of frequency operations and its properties are presented. Another subsection is devoted to the notations in process transfer functions in the MR topic, some elemental transformations between polynomials as well as the available relations between fast-skipped and slow or slow-expanded and fast signals. Finally the discrete lifting, traditionally introduced in an internal representation way, is adapted to our algebra. It is a section that is a survey to follow the design procedure in Section 3. First of all, it must be noted that the systems this contribution deals with are known as MultiMate Systems, that is, systems where there are sampled or discrete signals referred to two or more different frequencies. An initial scheme could help to understand different issues related to this kind of systems (see Figure 1).

One option in order to describe the different signals and systems in these environments is to use notation with superscripts. The signal (or system, when it is the case) *Y^T^* denotes either the *Z*-transform of the sequence *y*(*kT*) derived from the sampling with period *T* of the continuous signal *y*(*t*):

(1) $$Y^{T}≜Z\{y(k)\}=\overset{\infty }{\underset{k=0}{\text{∑}}}y(kT)z^{-k}$$

or the sampling rate transformation of a discrete signal *Y* (as will be explained below). With respect to Figure 1:

(2) $$Y^{NT}={\left[G(s)U^{MT}\right]}^{NT}=G^{NT}{\left[U^{MT}\right]}^{NT}$$

where *G^NT^* represents the continuous process discretization (usually ZOH-discretization) at period *NT*:

(3) $$G^{NT}=Z\left[\frac{1-e^{-\text{NTs}}}{s}G(s)\right]$$

This single example enables one to understand that the sampling period transformation between discrete signals or the sampling operations involving blocks of different nature is quite common in MR systems. One way to achieve a proper handling of this kind of systems requires management of the great common divisor (gcd) and the least common multiple (lcm) of every sampling period occurring in the studied MR scheme. With these magnitudes, every sampling period is going to be repeated an integer multiple of times in one lcm, *T*_0_. There also will be a base period (gcd), *T_B_* such that *T*_0_ = *PT_B_*, being *P* an integer greater than one. With respect to Figure 1, if it is called *T_0_* the lcm period and *B* = lcm (M,N), *T_B_* = *T*_0_/*B*.

Once the general MR problem has been introduced, the multirate input control case (MRIC), that is when the output sampling period is strictly greater than the input sampling period, will be assumed in the rest of this work. In these conditions, it is adequate to assume two sampling periods, input *T* and output *NT*, being *N* a positive integer number greater than *1* if a MR system is studied (*N* is known as multiplicity).

### 2.1. Signals: Basic MR Operations and Properties

Some operators adapted for the transformation between periods with integer multiplicity must be introduced. In this sense, it is useful to consider basic operations such as “skip” and “expand”, introduced by [7], that look at the data in the same way as the classical downsampling and upsampling operations used in signal processing.

If the *Z*-transform referred to period *NT* is defined as:

(4) $$Y^{NT}≜Z_{NT}\;\{y(k)\}=\overset{\infty }{\underset{k=0}{\text{∑}}}y(\text{kNT})z^{-kN}$$

it is possible to formally express:

- the *expand* (upsampling) operator creates a *T*-sequence from a *NT*-sequence, as follows:
  (5a) $${\left[Y^{NT}(z_{N})\right]}^{T}≜{Ῡ}^{T}(z^{N})≜\overset{\infty }{\underset{k=0}{\text{∑}}}\bar{y}(kT)z^{-kN};\{\begin{array}{c} \bar{y}(kT)=y(kT);\forall k=\lambda N\\ \bar{y}(kT)=0;\forall k\neq \lambda N \end{array}\lambda \in Z^{+}$$
- the skip (downsampling) operator creates a *NT*-sequence from a *T*-sequence, as follows:
  (5b) $${\left[Y^{T}(z)\right]}^{NT}≜{\overset{⌣}{Y}}^{NT}(z^{N})≜\overset{\infty }{\underset{k=0}{\text{∑}}}y(\text{kNT})z^{-kN}=Y^{NT}(z_{N})$$

**Figure 2.**
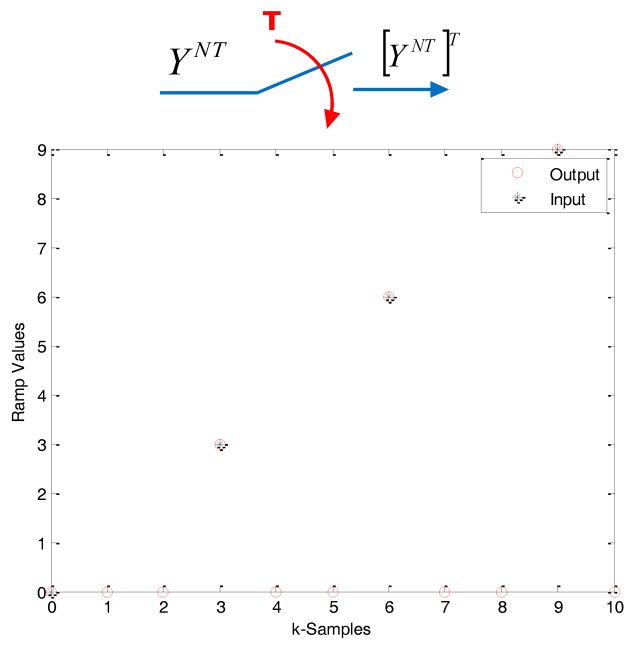
Expand operation. Case *N* = 3.

**Figure 3.**
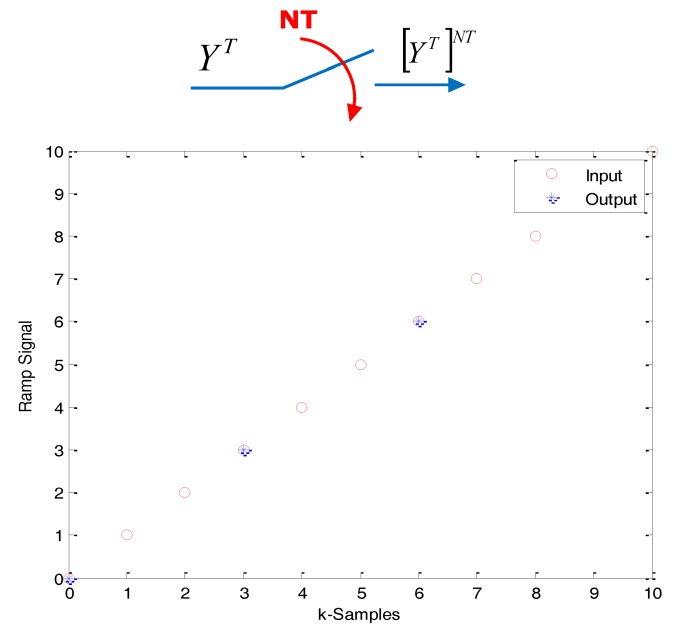
Skip operation. Case *N* = 3

For a graphical explanation of this kind of operators, see Figures 2 and 3.

The skip operation applied to the *Z*-transform of a signal can be obtained using the expression (6), due to Sklansky [8]:

(6) $${{\left[F^{T}(z)\right]}^{NT}=\frac{1}{N}\overset{N-1}{\underset{k=0}{\text{∑}}}F(\begin{array}{cc} e^{j\frac{2\pi k}{N}} & z) \end{array}|}_{z^{N}=z_{N}}={\overset{⌣}{F}}^{NT}(z_{N})$$

Some known skip-expand properties usually considered in this work are:

(7a) $${\left[X^{T}(z)Y^{T}(z)\right]}^{NT}\neq {\left[X^{T}\right]}^{NT}{\left[Y^{T}\right]}^{NT}(z_{N})$$

(7b) $${\left[X^{NT}(z_{N})Y^{NT}(z_{N})\right]}^{T}={\left[X^{NT}\right]}^{T}{\left[Y^{NT}\right]}^{T}(z^{N})$$

(7c) $${\left[X^{T}(z){\left[Y^{NT}\right]}^{T}(z)\right]}^{NT}={\left[X^{T}\right]}^{NT}Y^{NT}(z_{N})$$

that is, (a) *the skip does not commute*, and (b) *the expand commutes*. The third is a clear rule with an easy proof that will be used.

As an example guide to demonstrate every other property, the pattern “skip operation does not commute”, (7a), is proved. Without loss of generality *N* = *2* has been considered:

(8) $$\begin{array}{l} {\left[X^{T}Y^{T}\right]}^{2T}={\left\{\left[\overset{\infty }{\underset{k=0}{\text{∑}}}x(kT)z^{-k}\right]\;\left[\overset{\infty }{\underset{k=0}{\text{∑}}}y(kT)z^{-k}\right]\right\}}^{2T}=\\ ={\left[\left\{x_{0}+x_{1}z^{-1}+x_{2}z^{-2}+x_{3}z^{-3}+x_{4}z^{-4}+\ldots \}*\{y_{0}+y_{1}z^{-1}+y_{2}z^{-2}+y_{3}z^{-3}+y_{4}z^{-4}+\ldots \right\}\right]}^{2T}=\\ =[\{x_{0}y_{0}+(x_{1}y_{0}+x_{0}y_{1})z^{-1}+(x_{0}y_{2}+x_{1}y_{1}+x_{2}y_{0})z^{-2}+(x_{0}y_{3}+x_{1}y_{2}+x_{2}y_{1}+x_{3}y_{0})z^{-3}+\\ {(x_{0}y_{4}+x_{1}y_{3}+x_{2}y_{2}+x_{3}y_{1}+x_{4}y_{0})z^{-4}+\ldots ]}^{NT}=x_{0}y_{0}+(x_{0}y_{2}+x_{1}y_{1}+x_{2}y_{0})z^{-2}+\\ +(x_{0}y_{4}+x_{1}y_{3}+x_{2}y_{2}+x_{3}y_{1}+x_{4}y_{0})z^{-4}+\ldots \\ \neq {\left[X^{T}\right]}^{2T}{\left[Y^{T}\right]}^{2T}=\{x_{0}+0z^{-1}+x_{2}z^{-2}+0z^{-3}+x_{4}z^{-4}+\ldots \}*\{y_{0}+0z^{-1}+y_{2}z^{-2}+0z^{-3}+y_{4}z^{-4}+\ldots \}=\\ =\{x_{0}y_{0}+0z^{-1}+(x_{2}y_{0}+x_{0}y_{2})z^{-2}+0z^{-3}+(x_{4}y_{0}+x_{0}y_{4}+x_{2}y_{2})z^{-4}+\ldots \} \end{array}$$

Once some basic operations have been introduced, it is also very interesting to expose the application over transfer functions. From here, the expressions will be managed without the subindex “1” for the variable *z* (*T*-period). Every formula can be assumed in *z* referred to *T*, or *z_N_* = *z^N^* with respect to *NT*; the user knows the argument of every signal.

### 2.2. Process Transfer Functions: Notations and Transformations

Assuming the continuous time process in Figure 1, for any pair of sequences like those in that Figure, which are respectively considered as process output and input, a discrete time (DT) transfer function of the process plus the hold device can be written:

1. The fast sampling DT (FSDT) model is defined by:
  (9) $$\frac{Y^{T}(z)}{U^{T}(z)}=G^{T}(z)\equiv {\left[H_{T}G_{p}(s)\right]}^{T}=\frac{B^{T}(z)}{A^{T}(z)}=\frac{\begin{array}{cc} {\text{∑}}_{i=1}^{n}b_{i,T} & z^{-i} \end{array}}{\begin{array}{cc} 1+{\text{∑}}_{i=1}^{n}a_{i,T} & z^{-i} \end{array}}$$
  where *B^T^*(*z*), *A^T^*(*z*) are polynomials in *z*^−1^. Following the notation in (1), these polynomials also represent finite sequences with *n*-elements *b_i,T_*, *a_i,T_*, respectively, for *i* = 1, …, *n*, being *a*_0,_*_T_* = 1, and *b*_0,_*_T_* = 0. The expand and skip operators, (5), can also be applied to polynomials in *z*^−1^.
2. For the same process, a slow sampling DT (SSDT) model can be similarly defined by:
  (10) $$\frac{Y^{NT}(z^{N})}{U^{NT}(z^{N})}=G^{NT}(z^{N})\equiv {\left[H_{NT}G_{p}(s)\right]}^{NT}=\frac{B^{NT}(z_{N})}{A^{NT}(z_{N})}=\frac{\begin{array}{cc} {\text{∑}}_{i=1}^{n}b_{i,NT} & z^{-iN} \end{array}}{\begin{array}{cc} 1+{\text{∑}}_{i=1}^{n}a_{i,NT} & z^{-iN} \end{array}}$$
  where *B^NT^*(*z_N_*),*A^NT^*(*z_N_*) are polynomials in $z_{N}^{-1}$, and mathematically in *z*^−^*^N^*. The proper treatment expressed when defining the skip and expand signal operations will be assumed for these polynomials.

When the operation [•]*^T_i_^* is applied to a system it must be considered the *Z* transform of the impulse response of that system.

The FSDT transfer function poles are denoted by *α_i,T_*. That is:

(11) $$A^{T}(z)=\prod_{i=1}^{n}\left(z-\alpha_{i,T}\right)$$

If the SSDT poles are *α_i_*_,_*_NT_*, such that:

(12) $$A^{NT}(z^{N})=\prod_{i=1}^{n}\left(z^{N}-\alpha_{i,NT}\right)$$

Note that, dealing with the same continuous time system, *α_i_*_,_*_NT_* = *α_i_*_,_*_T_^N^*;∀*i* = 1,…,*n*.

Two technical assumptions are made:

- If α is a pole of *G^T^*(*z*), then $ae^{\overset{2\pi kj}{\;}/\underset{N}{\;}}$, *j*=*1*… (*N* − *1*),is not a pole of *G^T^*(*z*);
- All poles are different, *i.e.*, *α_i,T_* ≠ *α_j,T_*;*i* ≠ *j*.

Assumption (a) is required to avoid aliasing, [9]. By assumption (b) the notation is simplified. The following useful relationship is easily derived:

(13) $$W_{A}^{T}(z)=\frac{\prod_{i=1}^{n}\left(z^{N}-\alpha_{i,NT}\right)}{\prod_{i=1}^{n}\left(z-\alpha_{i,T}\right)}=\frac{{\left[A^{NT}(z^{N})\right]}^{T}}{A^{T}(z)}=\frac{{\bar{A}}^{T}(z^{N})}{A^{T}(z)}=\prod_{i=1}^{n}\left(z^{N-1}+\alpha_{i,T}z^{N-2}+\ldots +\alpha_{i,T}^{N-1}\right)$$

The FSDT model may be also expressed by:

(14) $$\frac{Y^{T}(z)}{U^{T}(z)}=G^{T}(z)=\frac{B^{T}(z)}{A^{T}(z)}=\frac{B^{T}(z)W_{A}^{T}(Z)}{A^{T}(z)W_{A}^{T}(Z)}=\frac{{\tilde{B}}^{T}(z)}{{\left[A^{NT}(z^{N})\right]}^{T}}=\frac{\begin{array}{cc} {\text{∑}}_{i=1}^{nN}{\tilde{b}}_{i,T} & z^{-i} \end{array}}{\begin{array}{cc} 1+{\text{∑}}_{i=1}^{n}a_{i,NT} & z^{-iN} \end{array}}$$

where *G^T^*(*z*) ≡ *Z_T_* [*H_T_*(*s*)*G_P_*(*s*)], being *Z_T_* the *Z* transform referred to a period *T* and *H_T_* a *T* zero order hold.

In what follows, some important results are proved:

From (14), it yields (every variable expressed in *z*):

(15) $${\tilde{B}}^{T}U^{T}={\left[A^{NT}\right]}^{T}Y^{T}$$

If a skip operation is applied to the *T*-time sequences, that is, doing a *NT* re-sampling, the result is:

(16) $${\left[{\tilde{B}}^{T}U^{T}\right]}^{NT}={\left[{\left[A^{NT}\right]}^{T}Y^{T}\right]}^{NT};{\left[{\tilde{B}}^{T}U^{T}\right]}^{NT}={\left[{\left[A^{NT}\right]}^{T}\right]}^{NT}{\left[Y^{T}\right]}^{NT};{\left[{\tilde{B}}^{T}U^{T}\right]}^{NT}=\left[A^{NT}\right]{\left[Y^{T}\right]}^{NT}$$

where [*A^NT^*]*^T^* is *A^NT^*, but expressed by means of the *z*-variable. The physical meaning is the consideration of a *NT* sampler at the process output, that is, a slow output:

(17) $${\left[{\tilde{B}}^{T}U^{T}\right]}^{NT}=A^{NT}Y^{NT}$$

In (17), it is not possible to isolate the elements of the term [*B̃^T^U^T^*]*^NT^*, because the skip operation does not commute, (7a). From this fact is derived that *it is not feasible to plan a transfer function between a skipped fast input and a slow output*.

The opposite situation is viable: the transformation of a slow frequency DT sequence into a fast frequency DT sequence. The dual rate zero order hold (DRZOH) device is defined by:

(18) $${\left[H_{NT}(s)\right]}^{T}=\frac{U_{O}^{T}}{{\left[U^{NT}\right]}^{T}}={\left[\frac{1-e^{-\text{NTS}}}{s}\right]}^{T}=\frac{1-z^{-N}}{1-z^{-1}}=(1+z^{-1}+\ldots +z^{-(N-1)})=:W_{R}^{-1}(z)=:{\left[W_{R}^{-1}\right]}^{T}$$

where the output is defined by $U_{O}^{T}$.

In this case, it is possible to obtain a transfer function of the process plus the DRZOH device:

(19) $$Y^{T}={\left[H_{NT}G_{P}(s)U^{NT}\right]}^{T}={\left[H_{NT}G_{P}(s)\right]}^{T}{\left[U^{NT}\right]}^{T}$$

because, as it is known, the expand operation commutes (7b). Also, using (18):

(20) $${\left[H_{NT}G_{P}(s)\right]}^{T}={\left[W_{R}^{-1}\right]}^{T}{\left[H_{T}G_{P}(s)\right]}^{T}$$

Thus, a dual rate discrete time (DRDT) operator is defined by:

(21) $$G^{T,NT}\equiv \frac{Y^{T}}{{\left[U^{NT}\right]}^{T}}={\left[W_{R}^{-1}\right]}^{T}G^{T}={\left[W_{R}^{-1}\right]}^{T}\frac{B^{T}}{A^{T}}={\left[W_{R}^{-1}\right]}^{T}\frac{B^{T}W_{A}^{T}}{A^{T}W_{A}^{T}}=\frac{{\left[W_{R}^{-1}\right]}^{T}{\tilde{B}}^{T}}{{\left[A^{NT}\right]}^{T}}$$

*G^T,NT^* describes the *transfer function from an expanded slow input* (*NT*) *to a fast output* (*T*).

Using a similar notation, the DRZOH operation, (18), can be represented by *H^T^*^,^*^NT^*. Clearly, it is also verified that:

(22) $${\left[\frac{Y^{T}}{{\left[U^{NT}\right]}^{T}}\right]}^{NT}=\frac{{\left[Y^{T}\right]}^{NT}}{{\left[{\left[U^{NT}\right]}^{T}\right]}^{NT}}=\frac{{\left[Y^{T}\right]}^{NT}}{U^{NT}}=\frac{{\left[{\left[W_{R}^{-1}\right]}^{T}{\tilde{B}}^{T}\right]}^{NT}}{{\left[{\left[A^{NT}\right]}^{T}\right]}^{NT}}=\frac{B^{NT}}{A^{NT}}$$

that is the SSDT model.

### 2.3. Discrete Lifting

When facing the modeling step of a MR system, most authors suggest the use of the so-called “Lifting” or “Discrete Lifting” method [10,11]. With this procedure every signal is referred to the lcm of the periods of the MR system, and consequently it is “lifted” at lcm period. This procedure can also be explained using the skip-expand operators. With the intention of showing the application of Lifting, a general MR scheme where *NT* is the lcm is assumed. One *T*-discrete signal *Y^T^* will be modeled in the lifted field by:

(23) $$Y_{l}(z^{N})=\left[\begin{array}{c} y_{l,0}(z^{N})\\ y_{l,1}(z^{N})\\ \vdots \\ y_{l,N-1}(z^{N}) \end{array}\right]=\left[\begin{array}{c} y_{0}+y_{N}z^{-N}+\ldots \\ y_{1}+y_{N+1}z^{-N}+\ldots \\ \vdots \\ y_{N-1}+y_{2N-1}z^{-N}+\ldots \end{array}\right]$$

That is, every sequence *y_l_*_,_*_i_*(*z^N^*) is obtained *expanding N* previously skipped elements from the *T*-original signal.

In this way it is possible to say that:

(24) $$Y(z)=\left(\begin{array}{ccc} 1 & z^{-1} & \begin{array}{ccc} z^{-2} & \ldots & z^{-(N-1)} \end{array} \end{array}\right)\cdot Y_{l}(z^{N})$$

because:

(25) $$y(z)=\overset{\infty }{\underset{k=0}{\text{∑}}}y_{k}z^{-k}=(y_{0}+y_{N}z^{-N}+\ldots )+z^{-1}(y_{1}+y_{N+1}z^{-N}+\ldots )+\ldots +z^{-(N-1)}(y_{N-1}+y_{2N-1}z^{-N}+\ldots )$$

It must be noted that *T* is the gcd period of the scheme.

Anyway, there are different options for Lifting application. All of them assume the technique of “Vector Switch Decomposition” [12]. That is, a multivariable system is achieved. It is usual to consider a state-space transformation, but one problem is derived of this. It is usual to find a mistake in some works that assume state-space control techniques; in the MR system there are not just variables with different dimensions, there are variables considered in different sampling times on one lcm period (sometimes called meta period or frame-period). That is the reason why in this contribution only the external representation will be considered [8,13–15].

### 2.4. Modeling MR Scheme at Fast Period

As exposed in Section 2.3, it is viable to express a fast signal as a sum of *N* slow signals. This result is especially interesting in order to solve the problem exposed in (17). In fact, using (7c):

(26) $$\begin{array}{l} {\left[G^{T}H^{T}\right]}^{NT}={\left[{\left(G_{0}^{NT}+z^{-1}G_{1}^{NT}+\ldots +z^{-(N-1)}G_{N-1}^{NT}\right)}^{T}H^{T}\right]}^{NT}=\\ {\left[{\left[G_{0}^{NT}\right]}^{T}H^{T}+z^{-1}{\left[G_{1}^{NT}\right]}^{T}H^{T}+\ldots +z^{-(N-1)}{\left[G_{N-1}^{NT}\right]}^{T}H^{T}\right]}^{NT}=\\ {\left[{\left[G_{0}^{NT}\right]}^{T}H^{T}\right]}^{NT}+{\left[{\left[G_{1}^{NT}\right]}^{T}z^{-1}H^{T}\right]}^{NT}+\ldots +{\left[{\left[G_{N-1}^{NT}\right]}^{T}z^{-(N-1)}H^{T}\right]}^{NT}=\\ G_{0}^{NT}{\left[H^{T}\right]}^{NT}+G_{1}^{NT}{\left[z^{-1}H^{T}\right]}^{NT}+\ldots +G_{N-1}^{NT}{\left[z^{-(N-1)}H^{T}\right]}^{NT} \end{array}$$

As it is proved, the terms can be separated. Actually, it is a laborious procedure but it assures the feasibility to get a fast sampling period modeling from a dual-rate closed loop, as it will be shown in Sections 3 and 4.

## 3. Problem Statement

**Figure 4.**
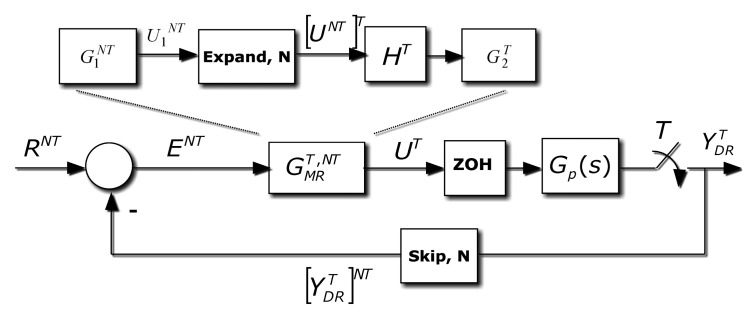
Dual rate controller structure.

The problem this contribution deals with appears when a computer control application, where the control algorithm implementation should be a *T* single rate control, is not viable. The restricted frequency sensor is the main reason. In different fields like chemical industries, artificial vision, network-based and distributed installations, the sensors need a certain amount of time (for computation requirements or due to sensor geographic location) that makes the ideal sampling period unfeasible. This is the environment where a MR control can be a valuable option. In order to overcome this problem, the idea is to reach the same *T*-behavior but measuring the controlled variable *N* times slower. As it easy to understand, a non-conventional controller is needed; the controller uses a slow input but it must deliver a fast output. The basic scheme is showed in Figure 4, where a dual-rate control is introduced. The plant is represented by an *n*-order single-input-single-output LTI continuous system (CT), with transfer function *G_p_*(*s*).

Based in this MRIC structure, that is slow measurement and fast control updating, in [8] a new non-conventional structure composed by slow and fast parts for the dual rate controller was exposed; an expand operation is required to assure the composition of two different frequency elements. The rest of the closed loop performs as follows: the controller output is updated at a period *T* through the fast hold device, *H^T^*, and the system output, *y*(*t*), is measured at period *NT*, being represented by a fast sampler followed by an *N*-sampler skip operation, and compared to the reference *R*(*t*) sampled at the slow rate, *R^NT^*. The dual rate controller, $G_{MR}^{T,NT}$, processes the error at slow rate, *E^NT^*, and generates *N* fast control actions, *U^T^*, inside the meta period *NT*. It must be noted that just the case where in all blocks input and output sampling periods are integer is considered; that is, the more complex case when rational ratio appears is out of our scope.

As Figure 4 shows, and using the skip-expand properties (7), the controller output can be given by:

(27) $$U^{T}=G_{2}^{T}{\left[U_{1}^{NT}\right]}^{T}=G_{2}^{T}{\left[G_{1}^{NT}\right]}^{T}{\left[E^{NT}\right]}^{T}=G_{2}^{T}{\left[G_{1}^{NT}\right]}^{T}{\left[R^{NT}-{\left[y_{DR}^{T}\right]}^{NT}\right]}^{T}$$

therefore:

(28) $$y_{DR}^{T}=G_{p}^{T}H^{T}G_{2}^{T}{\left[G_{1}^{NT}\right]}^{T}{\left[R^{NT}-{\left[y_{DR}^{T}\right]}^{NT}\right]}^{T}$$

and, since $y_{DR}^{T}\neq {\left[{\left[y_{DR}^{T}\right]}^{NT}\right]}^{T}$, the Equation (28) cannot be expressed in a closed form.

Nevertheless, if the dual rate modelling is considered at slow rate:

(29) $${\left[y_{DR}^{T}\right]}^{NT}={\left[G_{p}^{T}H^{T}G_{2}^{T}{\left[G_{1}^{NT}\right]}^{T}{\left[R^{NT}-{\left[y_{DR}^{T}\right]}^{NT}\right]}^{T}\right]}^{NT}={\left[G_{p}^{T}H^{T}G_{2}^{T}\right]}^{NT}G_{1}^{NT}\left[R^{NT}-{\left[y_{DR}^{T}\right]}^{NT}\right]$$

and finally:

(30) $$\frac{{\left[y_{DR}^{T}\right]}^{NT}}{R^{NT}}=\frac{{\left[G_{p}^{T}H^{T}G_{2}^{T}\right]}^{NT}G_{1}^{NT}}{1+{\left[G_{p}^{T}H^{T}G_{2}^{T}\right]}^{NT}G_{1}^{NT}}$$

With a similar procedure, the following expression is reached:

(31) $$\frac{y_{DR}^{T}}{{\left[R^{NT}\right]}^{T}}=\frac{G_{p}^{T}H^{T}G_{2}^{T}{\left[G_{1}^{NT}\right]}^{T}}{1+{\left[{\left[G_{p}^{T}H^{T}G_{2}^{T}\right]}^{NT}\right]}^{T}{\left[G_{1}^{NT}\right]}^{T}}$$

In order to avoid the denominator complexity; (26) could be used.

These expressions are useful when the simulation tool is implemented. Once the dual-rate closed loop modeling has been established (30), the design is faced. The goal is to force the skipped fast output, ${\left[Y_{DR}^{T}\right]}^{NT}$, to be the same than the slow single rate control loop output, *Y̅^NT^*, that is, [*M^T^R^T^*]*^NT^* should match to *M^NT^R^NT^*. One of the options is to consider a model based controller. In this way, if *M*(*s*) is considered as the reference model for the controlled system and *M^NT^* and *M^T^* are the ZOH discrete equivalent transfer functions from *M*(*s*), according to [8] the dual rate controller will be formed by:

- a fast part given by:
  (32) $$G_{2}^{T}(z)=\frac{M^{T}(z)}{{G_{P}}^{T}(z)}$$
- a slow part given by:
  (33) $$G_{1}^{NT}(z_{N})=\frac{1}{1-M^{NT}(z_{N})}$$
- a rate converter with the form:
  (34) $$H^{T}(z)=\frac{R^{T}(z)}{{\left[R^{NT}\right]}^{T}(z)}$$

It is easy to observe that the dual rate controller is tuned for one type of command signal *R*(*t*), since the rate converter (34) depends on the selected reference. In this paper this particular kind of rate converter has been used, but others can be used.

### 3.1. Model Based Controller

In this case, the design procedure is based on a continuous closed loop including a controller *G_R_*(*s*) (usually, a PID-type controller). From this controller a closed loop transfer function *M*(*s*) is obtained. Then, ZOH-discretization assuming periods *T* and *NT* must be computed in order to obtain *M^T^* and *M^NT^*, and respectively. So, applying (32)–(34) the design step is completed.

If the process is non-minimum phase, the cancellation of unstable pole-zero pairs must be avoided. Thus, the fast part of the controller could be alternatively computed by (35):

(35) $$G_{2}^{T}(z_{1})=\frac{M^{T}(z_{1})}{G^{T}(z_{1})}=\frac{G_{R}^{T}(z)}{1+G_{R}^{T}(z)G^{T}(z)}$$

If the slow part (33) is conserved, the design method is obviously not the introduced one, that is, the output does not match that predicted by the closed loop transfer function.

### 3.2. Cancellation Controllers

From the continuous time closed loop, it is possible to follow another design method. If some desired *M*(*s*) is considered (note that now *M*(*s*) is not derived from a closed loop containing a specific controller and plant), the expressions (32)–(34) give again the dual-rate controller. Obviously, it is a classical cancellation-type controller, and therefore the basic rules to select a proper *M*(*s*) must be assumed. For more information about different kinds of multi-rate cancellation controllers (Minimum Time, Finite Time), reader is referred to [8] and [16].

### 3.3. PID Dual Rate Controllers

With the proposed structure (28)–(30), another alternative way to obtain the slow and fast controller parts can be starting from a PID controller but without reference model. When a PID-type controller is considered, it is logical to think with the derivative action working in high frequencies and the integral action in the lower ones. Therefore, a possible decomposition according to classical discretization methods, [17] could be defined by:

(36) $$G_{PI}(z_{N})=K_{PI}\frac{z_{N}-\left(1-\frac{NT}{T_{i}}\right)}{z_{N}-1}$$

(37) $$G_{PD}(z)=K_{PD}\frac{z\left(1+\frac{T_{d}}{T}\right)-\frac{T_{d}}{T}}{z}$$

For more information, reader is referred to [18].

### 3.4. RST Controllers

**Figure 5.**
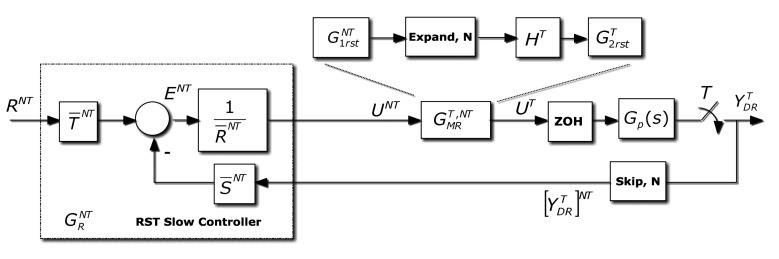
Block diagram for the control system: multi-rate controller including an RST stage.

The RST controller [19] is a two-degree-of-freedom controller. Its design is an input-output model-based pole placement design procedure, requiring the resolution of a Diophantine equation. In this paper (as shown in Figure 5) to avoid confusion, the *RST* polynomials will be named as *R̅S̅T̅* (since *R* is used for the reference signal, and *T* for the sampling period). The polynomials *R̅S̅* are deduced when solving the Diophantine equation. The polynomial *T̅* is designed to tune the control system gain, and to avoid unstable zero cancellations.

In this case, *R̅S̅T̅* control law is designed (see [1]) at period *NT*, and yields:

(38) $${\bar{U}}^{NT}=\frac{{\bar{T}}^{NT}(z_{N})}{{\bar{R}}^{NT}(z_{N})}R^{NT}-\frac{{\bar{S}}^{NT}(z_{N})}{{\bar{R}}^{NT}(z_{N})}{\left[Y_{DR}^{T}\right]}^{NT}$$

This slow frequency control signal is modified by a dual rate part obtained from (32)–(34). So, from:

(39) $$M^{NT}=\frac{G_{\text{RST}}^{NT}G^{NT}}{1+G_{\text{RST}}^{NT}G^{NT}}$$

the slow side of the multi-rate controller can be rewritten in this way:

(40) $$\frac{1}{1-M^{NT}}=G_{\text{RST}}^{NT}\frac{G^{NT}}{M^{NT}}$$

Thus, the multi-rate controller can be redefined as follows:

(41) $$G_{MR}^{T,NT}=\frac{M^{T}}{G^{T}}\frac{R^{T}}{{\left[R^{NT}\right]}^{T}}{\left[G_{\text{RST}}^{NT}\frac{G^{NT}}{M^{NT}}\right]}^{T}$$

That is using (13):

(42) $${\left[G_{1\text{rst}}^{NT}\right]}^{T}={\left[\frac{G^{NT}}{M^{NT}}\right]}^{T}=\frac{{\left[B^{NT}\right]}^{T}}{{\left[A^{NT}\right]}^{T}}\frac{{\left[A_{M}^{NT}\right]}^{T}}{{\left[B_{M}^{NT}\right]}^{T}}=\frac{{\left[B^{NT}\right]}^{T}}{{\left[B_{M}^{NT}\right]}^{T}}\frac{A_{M}^{T}W_{M_{A}}^{T}}{A^{T}W_{A}^{T}}$$

In addition, $G_{2\text{rst}}^{T}(z)$ can be represented by:

(43) $$G_{2\text{rst}}^{T}=\frac{M^{T}}{G^{T}}=\frac{B_{M}^{T}}{A_{M}^{T}}\frac{A^{T}}{B^{T}}$$

Leading to:

(44) $$G_{2\text{rst}}^{T}{\left[G_{1\text{rst}}^{NT}\right]}^{T}=\frac{B_{M}^{T}}{A_{M}^{T}}\frac{A^{T}}{B^{T}}\frac{{\left[B^{NT}\right]}^{T}}{{\left[B_{M}^{NT}\right]}^{T}}\frac{A_{M}^{T}(z)W_{M_{A}}^{T}}{A^{T}(z)W_{A}^{T}}=\frac{B_{M}^{T}W_{M_{A}}^{T}}{B^{T}W_{A}^{T}}\frac{{\left[B^{NT}\right]}^{T}}{{\left[B_{M}^{NT}\right]}^{T}}$$

which define the slow and fast parts of the dual rate controller. For more information, the reader is referred to [20].

### 3.5. Processes with Time Delay

In the MR control design field it is difficult to find contributions about processes with time-delay. The projected interactive simulation application tackles this problem, even in the general case when the delay is integer or non-integer with respect to the fast *T* and slow *NT* sampling periods in the dual-rate scheme. In general the delay, *d*, will be:

(45) d=L×NT+J×T+m

being *L* and *J* the integer multiplicities with respect to the slow and fast sampling periods.

Revisiting the control scheme already shown in Figure 4, in this case the Smith's Predictor (SP) proposal is adapted. Basically, this solution looks like a classical SP scheme, but now the novelty is provided by the algebra introduced in Section 2. In general, if *d* ≠ 0, the ZOH discretization of the process must be faced using the modified *Z*-transform [21].

With respect to the Figure 4, if the expression (28) is revisited and a skip operation is assumed:

(46) $${\left[Y^{T}\right]}^{NT}={\left[{\left(ZOH_{T}G_{p}(s)\right)}^{T}H^{T}G_{2}^{T}\right]}^{NT}G_{1}^{NT}E^{NT}$$

(47) $$E^{NT}=R^{NT}-{\left[Y^{T}\right]}^{NT}$$

If the process includes a transport delay *d*, then:

(48) $$\begin{array}{l} {\left[Y^{T}\right]}^{NT}={\left[{\left(ZOH_{T}G_{p}(s)e^{-ds}\right)}^{T}H^{T}G_{2}^{T}\right]}^{NT}G_{1}^{NT}E^{NT}={\left[{\left(ZOH_{T}G_{p}(s)e^{-(L\times NT+J\times T+m)s}\right)}^{T}H^{T}G_{2}^{T}\right]}^{NT}G_{1}^{NT}E^{NT}=\\ {\left[z^{-LN}z^{-J}{\left(ZOH_{T}G_{p}(s)e^{-ms}\right)}^{T}H^{T}G_{2}^{T}\right]}^{NT}G_{1}^{NT}E^{NT}=z^{-L}{\left[z^{-J}{\left(ZOH_{T}G_{p}(s)e^{-ms}\right)}^{T}H^{T}G_{2}^{T}\right]}^{NT}G_{1}^{NT}E^{NT} \end{array}$$

That is, the branch to subtract in order to avoid the effect of the delay.

**Figure 6.**
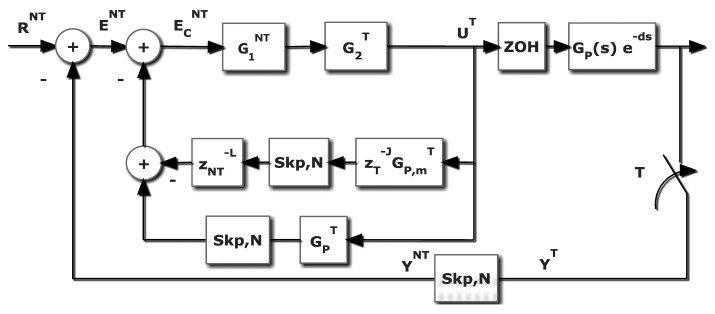
Dual rate scheme with Smith's Predictor.

**Figure 7.**
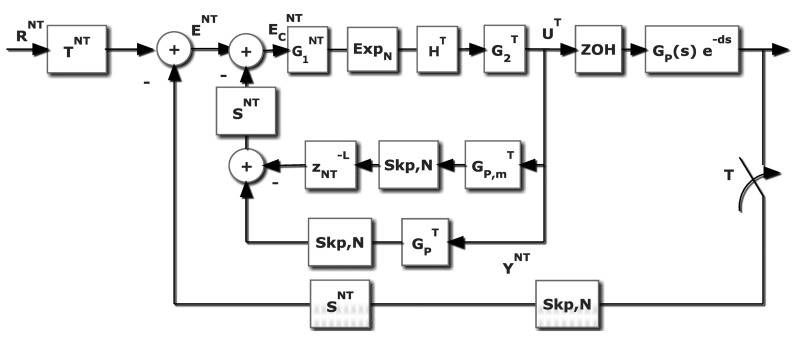
RST Controller in a loop with dead time.

It must be noted that (*ZOH_T_G_p_*(*s*) *e*^−^*^ms^*)*^T^* represents the process with *T*-zero order hold *Z*-modified transform. In Figure 6, $G_{p,m}^{T}$ represents the *T*-ZOH discretization with a delay (*J* × *T* + *m*); obviously if *m* ≠ 0, a *T*-modified *Z* transform must be considered. This procedure can be directly applied to the cancellation, model-based and PID controllers. In the case of RST, a special figure must be adapted (see Figure 7), but the proof is very similar.

## 4. Description of the Application

To better understand the multi-rate control aspects previously presented, an interactive simulation application has been developed. An executable version can be downloaded online from [22]. The main goal of this section is to detail the main features of this application, which has been programmed using an interactive simulation tool named SysQuake [6]. SysQuake uses a Matlab-like programing language, which is provided with some specific commands in order to perform a high level of interactivity in the developed application. This fact enables students to more easily learn about advanced, complex control concepts and techniques, and researchers to exploit and improve their achievements [23–25].

**Figure 8.**
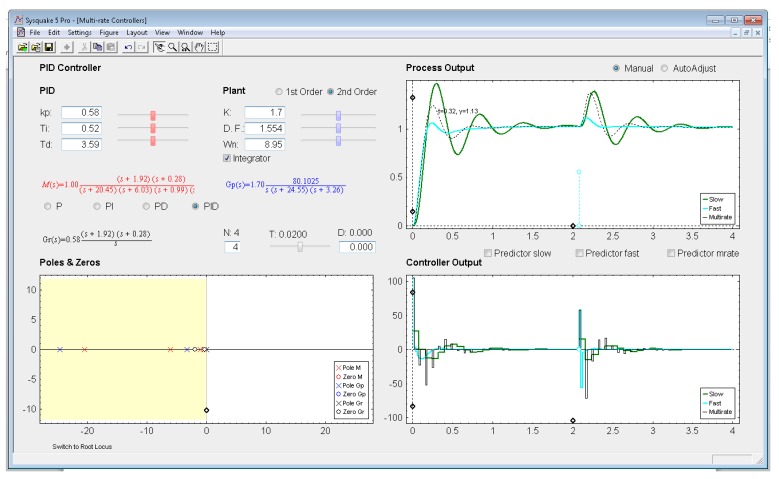
Application user interface for the PID controller.

Being aware interactivity is difficult to be shown in a writing text, next the different sections of the interactive application will be described. The application user interface presents a main window which is split into two parts (see in Figure 8): parameter section (upper left-hand part) and graphic section (lower left-hand part, and right-hand part). In addition, at the top side, the interface shows a menu bar and a toolbar, and at the bottom side, a status bar. These bars are provided by SysQuake and their working mode is standard, except for the *Settings menu*, which can be intentionally defined by the programmer.

**Figure 9.**
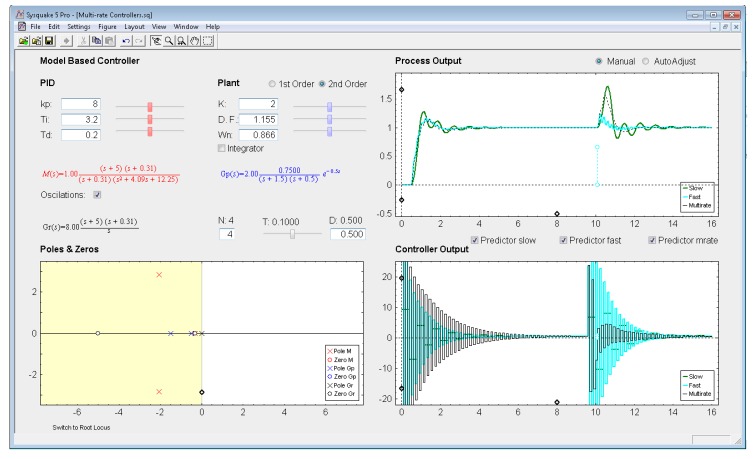
Application user interface for the Model-based (PID) controller.

**Figure 10.**
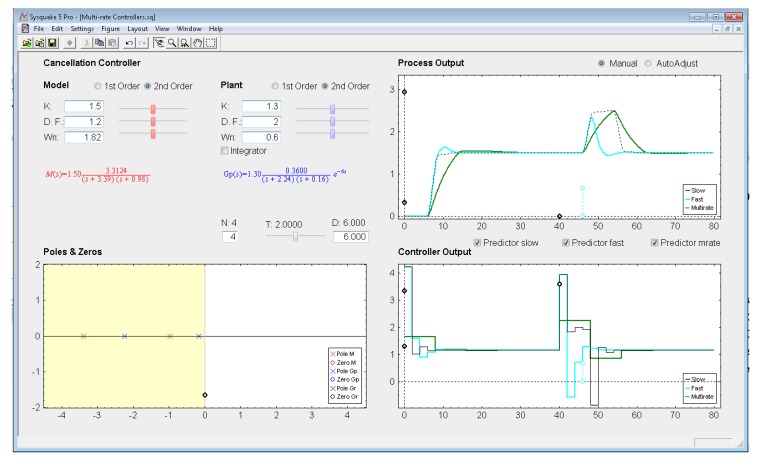
Application user interface for the Cancellation controller.

**Figure 11.**
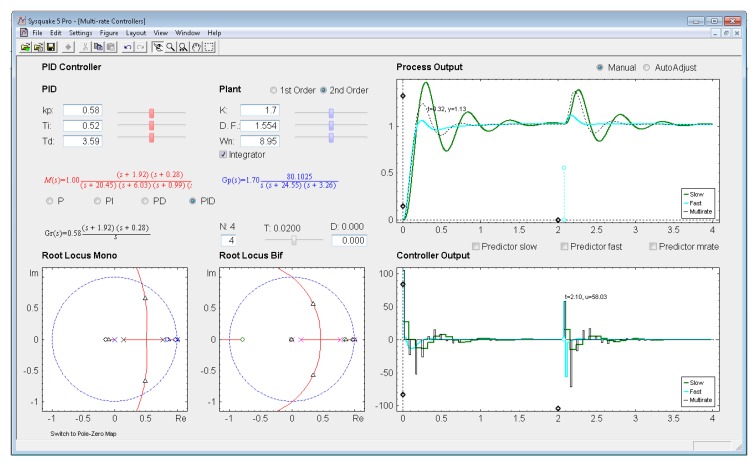
Slow-rate and dual-rate root locus graphic.

- *Settings menu:* by means of the option *Controller* of this menu, one of the four multi-rate controllers presented in the previous sections (PID controller, Cancellation controller, RST controller, or Model-based controller) can be chosen. Moreover, increments/decrements for every slider included in the interface can be defined by means of the option *Slider Config* of this menu, which provides two variation modes for the sliders: by percentage or by fixed value.
- *Parameter section:* this section (located in the upper left-hand part; see Figure 8) enables to introduce the input data required in each case. The section is split into two parts in order to consider input data of different nature: process parameters (right-hand part) and controller parameters (left-hand part).
  Regarding the process parameters, two radio buttons enable selection of the process model complexity (first or second order). Data entries to define the consequent process parameters can be carried out via sliders or text boxes. Below these entries, a checkbox named *Integrator* is located, which can be activated to add an integrator to the selected model. In addition, the sampling time *T*, the multiplicity *N*, and the process time-delay *D* can be defined via slider (the first one) or text boxes (the last ones).While inserting every process parameter, the current process model is presented in blue text as a continuous-time transfer function *G_p_*(*s*) (below the checkbox).
  With respect to the controller parameters, the input data depend on the chosen controller. Thus, firstly, for PID controllers (illustrated in Figure 8), four radio buttons allow choosing the controller complexity (P, PI, PD, or PID), and consequently, the data entries for each basic control action can be carried out via sliders or text boxes. While inserting every parameter, the current continuous-time transfer function for the controller *G_r_*(*s*) appears in black text below the four radio buttons, whereas the closed-loop transfer function *M*(*s*) appears in red text above the radio buttons. Secondly, for Model-based controllers (see Figure 9), their interface is similar to the previous one, but, since only a PID controller structure can be treated, the four radio buttons are eliminated. Another difference is the existence of a checkbox named *Oscillations*, which can be deactivated to eliminate process output oscillations (remember (35)). Finally, for Cancellation and RST controllers (see Figure 10), a similar interface to that described when inserting the process parameters is used, but now with the goal of defining the desired continuous-time reference model *M*(*s*)(which, as previously, appears in red text as a transfer function). As a summary, Table 1 gathers every parameter employed in the developed application.
- *Graphic section:* controlled system output (named *Process Output*), control action signal (named *Controller Output*), and poles and zeroes map (named *Poles & Zeros*) are the figures plotted in this section in the main window. In addition, for PID and Model-based controllers, an option named *Switch to Root Locus* is available to replace the *Poles & Zeros* figure with the slow-rate and dual-rate root locus (named *Root Locus Mono* and *Root Locus Bif*, respectively; see Figure 11). When switching to this plot, a new option named *Switch to Pole-Zero Map* appears and enables to come back to the previous figure.

Regarding the *Process Output* and *Controller Output* figures, they show the simulation responses for the single-rate (slow-rate in green, and fast-rate in cyan) and the dual-rate case (in black), which are achieved by the chosen control algorithm when following a step reference. In these graphics, an impulse-like load disturbance can also be considered by dragging on the cyan circles to interactively define its amplitude and application time (at instant multiple of *NT*). Moreover, scales can be redefined by dragging or clicking on the black diamonds. Another graphic feature can be observed in Figure 11: if the mouse is positioned on the process output or on the controller output, a text appears indicating the time instant and the output value for this mouse position.

With respect to *Poles & Zeros* figure, it shows the location of the different continuous-time poles (crosses) and zeroes (circles) for the process (in blue), for the controller (in black; if PID or Model-based controllers are chosen), and for the closed-loop model (in red). Note that the colors are consequent with those used for the different transfer functions. By dragging on poles and zeroes the related continuous-time transfer functions and parameters change interactively, and *vice versa*. Finally, *Root Locus* figures show the discrete-time root locus for the slow-rate and dual-rate cases (observe that in this plot closed-loop poles appear and are represented by means of black triangles). Contrarily to the previous figure, poles and zeroes cannot be now dragged, but they change their location interactively when the appropriate parameters are modified.

## 5. Examples

In this section an example for each controller will be presented. Output responses for the single-rate and multi-rate cases will be compared and analyzed, focusing on multi-rate control benefits.

### 5.1. Model-Based Controller

Let us consider the example shown in Figure 9, where the process is defined by:

(49) $$G_{p}(s)=\frac{1.5}{\left(s+0.5)(s+1.5\right)}e^{-0.5s}$$

An acceptable continuous-time PID controller is given by *K_p_* = 8, *T_D_* = 0.2, *T_I_* = 3.2 (considering $u(t)=K_{P}\left[e(t)+T_{D}e(t)+\frac{1}{T_{I}}\int_{0}^{t}e(\tau )d\tau \right]$), which yields:

(50) $$G_{r}(s)=8\frac{\left(s+5)(s+0.31\right)}{s}$$

**Figure 12.**
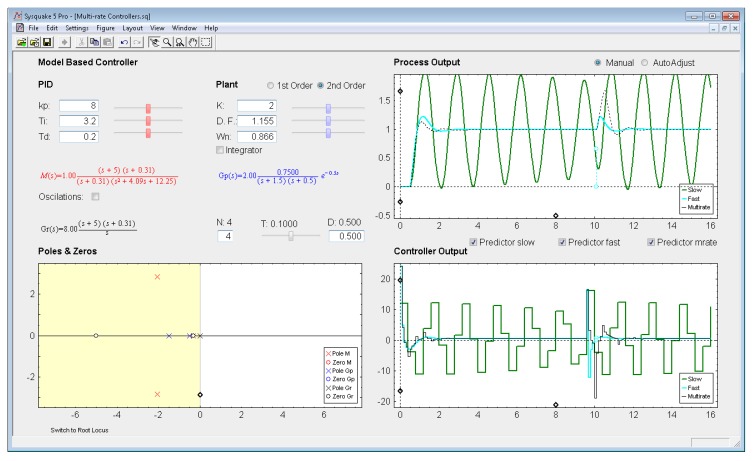
Application user interface for the Model-based (PID) controller with no oscillations.

From (49)–(50) a continuous-time closed-loop transfer function *M*(*s*) can be calculated. Then, if the sampling time is *T* = 0.1 s, and the multiplicity is *N* = 4, the dual-rate controller is designed from (32)–(34) (note that the single-rate controllers can be designed using (32) and (33) with the specific rate for each controller). Responses for each case are illustrated in Figure 9, where stable outputs are always obsered due to the use of the *Predictor* options to face the process time delay (*D* = 0.5). In this example, the dual-rate controller is able to achieve a response which is quite similar to that obtained by the fast-rate controller when following a step reference. However, when applying a load disturbance, the dual-rate output worsens with respect to the fast-rate one, since the dual-rate controller is designed for step references and not for impulse inputs like the load disturbance is. Regarding the slow-rate output, it is clearly worse for the both cases (when following a step reference and when applying a load disturbance), since it shows around 10% higher overshoot and 2 times longer settling time with respect to the dual-rate output. In any case, every output shows oscillations (ripple) due to cancelling the process dynamics. Deactivating the checkbox *Oscillations*, the controllers are designed following (35), and then the ripple effect totally disappears (see Figure 12). Moreover, in this case, whereas the slow-rate output is on the verge of instability, the dual-rate output presents around 5% less overshoot than the fast-rate output when following a step reference.

### 5.2. Cancellation Controller

Let us consider the example shown in Figure 10, where the process is defined by:

(51) $$G_{p}(s)=\frac{0.47}{\left(s+2.24)(s+0.16\right)}e^{-6s}$$

In order to decrease around 10 times the settling time, the next closed-loop transfer function is chosen:

(52) $$M(s)=\frac{4.97}{\left(s+3.39)(s+0.98\right)}$$

If the sampling time is *T* = 2 s and the multiplicity is *N* = 4, as in the previous example a different controller can be designed using (32)–(34). Responses for each case are illustrated in Figure 10, where as a consequence of including the *Predictor* options in every control algorithm, every response is stable. In this example, the dual-rate controller is also able to achieve a better response (no overshoot and around 20% shorter settling time) than that obtained by the fast-rate controller when following a step reference. However, as previously, when applying a load disturbance, the dual-rate output worsens with respect to the fast-rate one (around 10% higher overshoot, and around 15% longer settling time). Regarding the slow-rate output, it is clearly worse considering the settling time index (around 50% longer with respect to the dual-rate output). The overshoot index is very similar.

### 5.3. PID Controller

Let us consider the example shown in Figure 8, where the process is defined by:

(53) $$G_{p}(s)=\frac{136.17}{s(s+24.55)(s+3.26)}$$

An acceptable continuous-time PID controller is given by *K_p_* = 0.58, *T_D_* = 0.3.59, *T_I_* = 0.52 (considering $u(t)=K_{P}\left[e(t)+T_{D}e(t)+\frac{1}{T_{I}}\int_{0}^{t}e(\tau )d\tau \right]$). From (31)–(32), and assuming *K*_p1_ = 1 and *K*_PD_ = *K*_p_, and *T* = 0.02 s and *N* = 4, the dual-rate controller is designed (note that the single-rate controllers can be designed using (36) and (37) with the specific rate for each controller). Responses for each case are illustrated in Figure 8.

**Figure 13.**
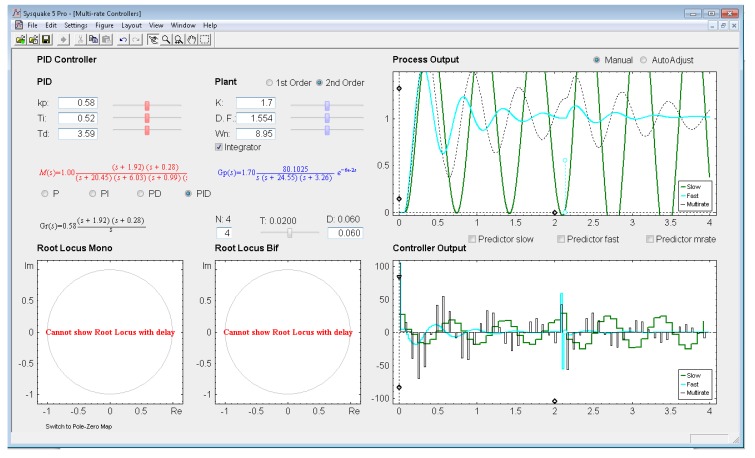
Application user interface for the PID controller with process time delay and no predictor.

**Figure 14.**
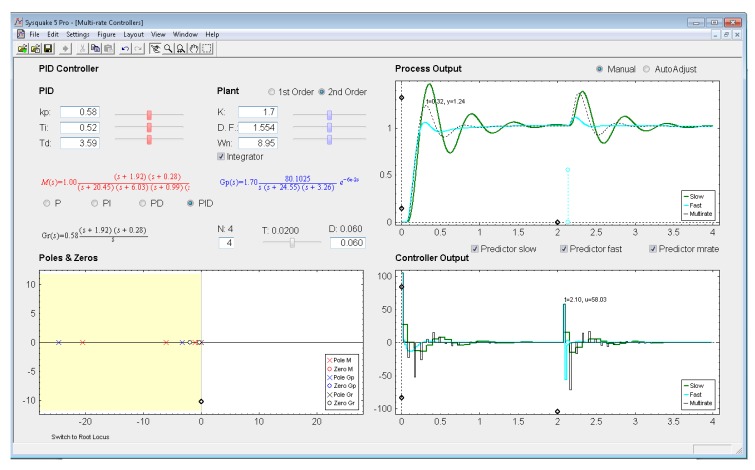
Application user interface for the PID controller with process time delay and predictors.

In this example, the dual-rate response reaches time indexes whose values are between those obtained by the single-rate controllers. If some process time delay were considered, for example *D* = 0.06, the responses would significantly worsen (concretely, the slow-rate one would become unstable), as depicted in Figure 13 (where every *Predictor* option is now deactivated). In addition, and as well-known, the different root locus could not be generated. Nevertheless, if the *Predictor* options were activated, every response would recover the previous behavior (see Figure 14).

### 5.4. RST Controller

Let us consider the next example, where the process is defined by:

(54) $$G_{p}(s)=\frac{128.62}{{\left(s+2.42\right)}^{2}+11.08^{2}}$$

In order to eliminate the overshoot and slightly decrease the settling time, the next closed-loop transfer function is chosen:

(55) $$M(s)=\frac{60.72}{\left(s+26.12)(s+3.02\right)}$$

**Figure 15.**
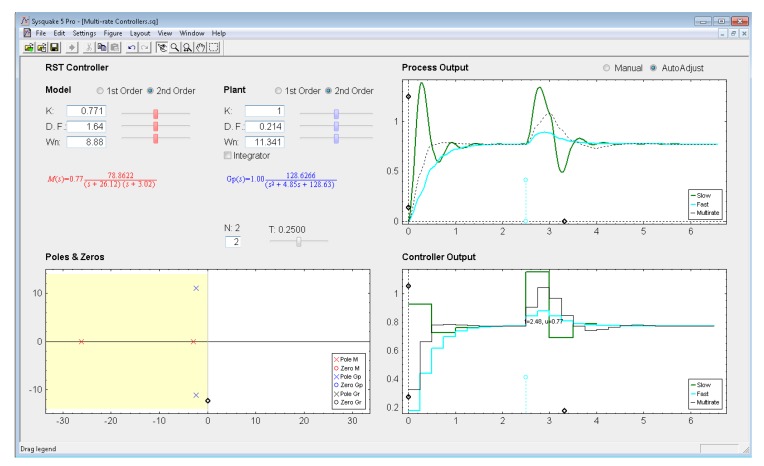
Application user interface for the RST controller.

If the sampling time is *T* = 0.25 s and the multiplicity is *N* = 2, from (42)–(43) the dual-rate controller is designed (the single-rate controllers can be designed from (41) considering the appropriate rate in each case). The different outputs are illustrated in Figure 15. Once again, the dual-rate controller is able to achieve a better response than the fast-rate one when following a step reference. Concretely, the settling time achieved is around twice as short as that obtained by the fast-rate controller. However, as previously, when applying a load disturbance, the dual-rate output worsens with respect to the fast-rate one, yielding around twice as much overshoot and settling time. Regarding the slow-rate output, it presents an overshoot clearly worse than the dual-rate one. The settling time is also worse when following a step reference but quite similar when applying a load disturbance.

## 6. Conclusions

One solution for the problem of control schemes with slow sensors is to assume a MR system, considering a restricted slow measurement sampling but also a faster control updating. the use of an interactive simulation tool in order to study multirate systems is a feasible option to make proper decisions about the correct control design. Different non-conventional controller structures have been provided. From application some unexpected results are achieved and explained. Even for an expert in this field the tool appears absolutely essential and time saving. For a beginner student or researcher it is entirely necessary if the study of this kind of systems is needed/desired.

## Figures and Tables

**Table 1.** Parameters available in the interactive simulation application.

**Parameter**	**Description**
kp, Ti, Td	PID proportional gain, integral time, and derivative time, respectively.
P, PI, PD, PID	Four radio buttons to select the PID-type controller complexity.
K, T.C.	Static gain and time constant, respectively, for a first order model/process.
K, D.F., Wn	Static gain, damping factor, and natural frequency, respectively, for a second order model/process.
1st Order, 2nd Order	Two radio buttons to select the model/process complexity.
Integrator	A checkbox to add an integrator to the process (when activated).
Oscillations	A checkbox to eliminate process output oscillations in Model-based controllers (when deactivated).
N	Multiplicity.
T	Sampling time.
D	Process time delay.
Switch to Root Locus	A link to show the discrete-time root locus plots.
Switch to Pole-Zero Map	A link to come back to the continuous-time poles and zeroes map.
Manual, AutoAdjust	Two radio buttons to select operation mode for figure scales.
Predictor slow	A checkbox to include a Smith's Predictor in the slow-rate control algorithm (when activated).
Predictor fast	A checkbox to include a Smith's Predictor in the fast-rate control algorithm (when activated).
Predictor mrate	A checkbox to include a Smith's Predictor in the multi-rate control algorithm (when activated).
